# Supplementary material for: Differentiation of Myocardial Properties in Physiological Athletic Cardiac Remodeling and Mild Hypertrophic Cardiomyopathy
Source: Biomedicines. 2024 Feb 12;12(2):420. doi: 10.3390/biomedicines12020420 (PMC10886585; doi:10.3390/biomedicines12020420)
Supplement: Supplementary file 1 [file biomedicines-12-00420-s001.zip › biomedicines-2822404-supplementary.pdf]

Table S1. Supplementary ROC analysis

	AUC	95% CI
**Echocardiography**		
E/A-ratio	0.77	0.62–0.90
E/e′	0.91	0.82–0.99
LV 3D end-diastolic volume	0.95	0.90–0.99
RV 2D fractional area change	0.78	0.66–0.91
**CMR**		
LGE %	0.71	0.55–0.89
LV ejection fraction	0.75	0.55–0.88
LV end-diastolic volume	0.95	0.90–0.99
RV ejection fraction	0.83	0.71–0.96
RV end-diastolic volume	0.96	0.91–1.00

Ability of indices from echocardiography and CMR to identify HCM mutation carriers from athletes in the study population evaluated by ROC analysis. A = late diastolic mitral flow velocity. AUC = area under the curve. CI = confidence interval. CMR = cardiac magnetic resonance imaging. E = early diastolic mitral inflow velocity. e′ = early diastolic mitral annular tissue velocity. HCM = hypertrophic cardiomyopathy. LGE % = late gadolinium enhancement extent expressed as percentage of the total left ventricular myocardium. LV = left ventricular. RV = right ventricular.
